# Defect Inspection Techniques in SiC

**DOI:** 10.1186/s11671-022-03672-w

**Published:** 2022-03-04

**Authors:** Po-Chih Chen, Wen-Chien Miao, Tanveer Ahmed, Yi-Yu Pan, Chun-Liang Lin, Shih-Chen Chen, Hao-Chung Kuo, Bing-Yue Tsui, Der-Hsien Lien

**Affiliations:** 1 Institute of Electronics, College of Electrical and Computer Engineering, National Yang Ming Chiao Tung University, Hsinchu, 30010 Taiwan; 2 Semiconductor Research Center, Hon Hai Research Institute, Taipei, 11492 Taiwan; 3 Department of Photonics and Institute of Electro-Optical Engineering, College of Electrical and Computer Engineering, National Yang Ming Chiao Tung University, Hsinchu, 30010 Taiwan; 4 Department of Electrophysics, College of Science, National Yang Ming Chiao Tung University, Hsinchu, 30010 Taiwan

**Keywords:** SiC, Killer defect, Defect inspection technology

## Abstract

With the increasing demand of silicon carbide (SiC) power devices that outperform the silicon-based devices, high cost and low yield of SiC manufacturing process are the most urgent issues yet to be solved. It has been shown that the performance of SiC devices is largely influenced by the presence of so-called killer defects, formed during the process of crystal growth. In parallel to the improvement of the growth techniques for reducing defect density, a post-growth inspection technique capable of identifying and locating defects has become a crucial necessity of the manufacturing process. In this review article, we provide an outlook on SiC defect inspection technologies and the impact of defects on SiC devices. This review also discusses the potential solutions to improve the existing inspection technologies and approaches to reduce the defect density, which are beneficial to mass production of high-quality SiC devices.

## Introduction

Owing to the rapid growth of power electronics market, SiC, a wide-bandgap semiconductor, emerges as a promising candidate to develop the next-generation power devices used in electric vehicles [1], aerospace [2] and power conversion applications [3, 4]. SiC-based power electronics offers several advantages over conventional devices made from Si or GaAs. Table 1 shows the physical properties of SiC compared with those of Si and GaAs as well as other wide-bandgap materials, such as GaN and diamond. Attributed to a wide bandgap (~ 3.26 eV for 4H-SiC), SiC-based devices can operate at higher electric fields and higher temperatures with a better reliability over Si-based power electronics. SiC also exhibits excellent thermal conductivity (about three times that of Si), which enables higher power density package for SiC devices with better heat dissipation. Its superior saturation electron velocity (about two times that of Si) allows for higher frequencies of operation with lower switching losses than Si-based power devices [4, 5]. The outstanding physical properties of SiC make it very promising for the development of a wide range of electronic devices, such as power MOSFETs with high blocking voltage and low on-resistance [6–8] as well as Schottky barrier diodes (SBD) that can withstand large breakdown fields with small reverse leakage currents [9].

**Table 1:** Physical properties (room temperature values) of wide-bandgap semiconductors for power electronic applications in comparison with conventional semiconducting materials [10–12]

Property	Silicon	3C-SiC	4H-SiC	GaAs	GaN	Diamond
Bandgap energy (eV)	1.1	2.2	3.26	1.43	3.45	5.45
Breakdown field (10^6^ Vcm^−1^)	0.3	1.3	3.2	0.4	3.0	5.7
Thermal conductivity (Wcm^−1^ K^−1^)	1.5	4.9	4.9	0.46	1.3	22
Saturated electron velocity (10^7^ cm s^−1^)	1.0	2.2	2.0	1.0	2.2	2.7
Electron mobility (cm^2^V^−1^ s^−1^)	1500	1000	1140	8500	1250	2200
Melting point (°C)	1420	2830	2830	1240	2500	4000

**Fig. 1:**
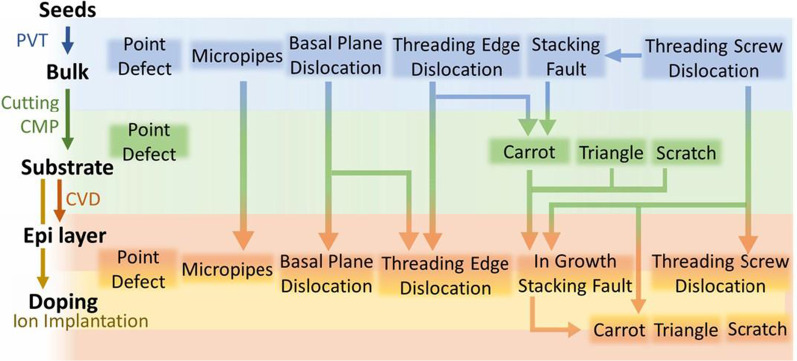
Schematic diagram of SiC growth process and various kind of defects caused by each step

Improving SiC wafer quality is important for manufacturers as it directly defines the performance of SiC devices and thus, dictates the production cost. However, the growth of SiC wafers with low defect density remains very challenging. Recently, the evolution of SiC wafer fabrication has accomplished a difficult transition from 100 mm (4-inch) to 150 mm (6-inch) wafer. SiC needs to be grown in a high-temperature environment along with its high rigidity and chemical stability, which leads to a high density of crystallographic and surface defects in the grown SiC wafer, resulting in poor quality of substrates and subsequently fabricated epitaxial layers. Figure 1 summarizes various kind of defects in SiC and the process steps which these defects originate from, and further discussion will be covered in the following section.

**Fig. 2:**
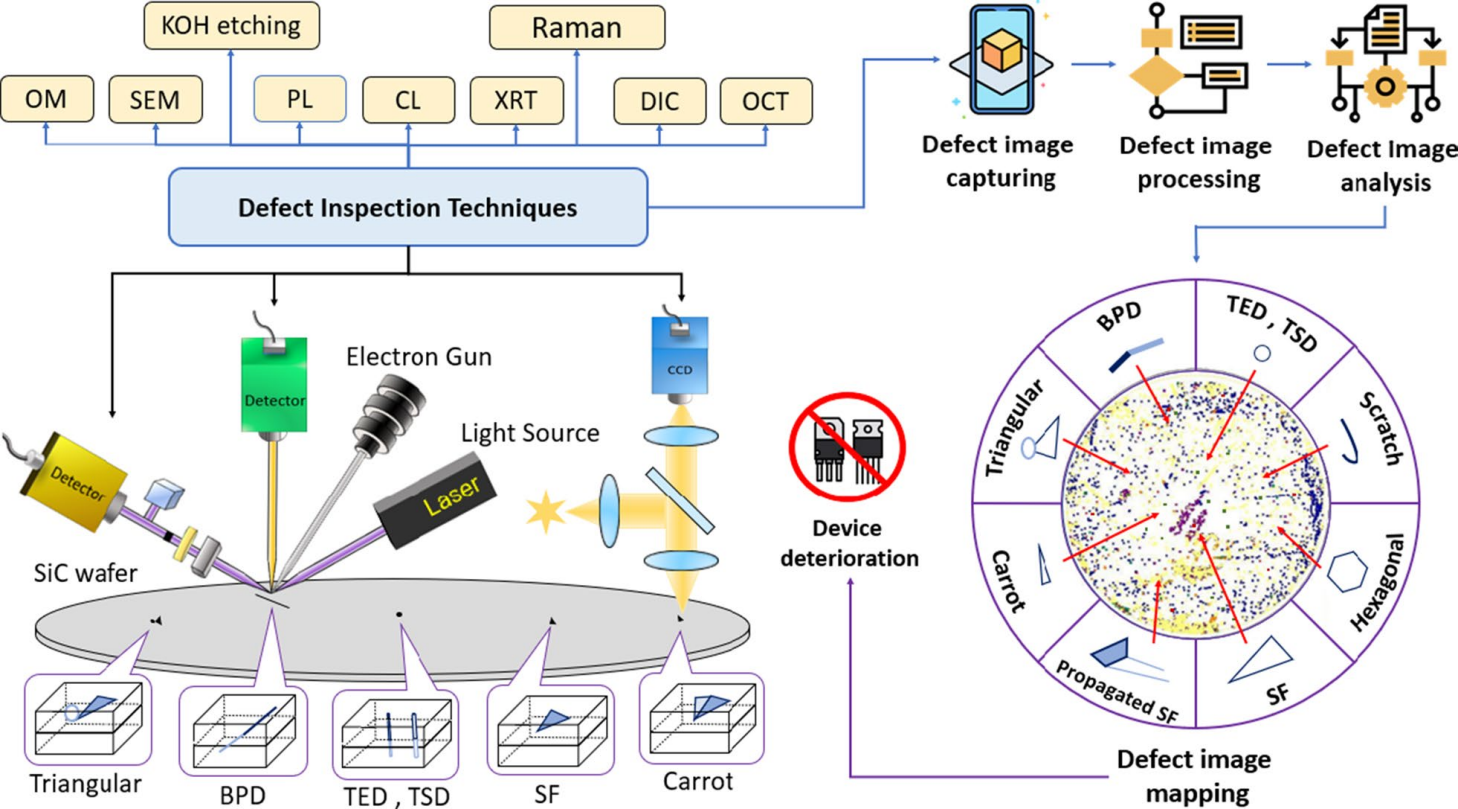
Available defect inspection technologies for SiC

Various types of defects cause different degrees of deterioration to device performance and may even lead to complete failure of the device. In order to improve the yield as well as the performance, the technology of inspecting defects prior to device fabrication becomes very important. Therefore, a rapid, highly accurate, and non-destructive inspection technology plays an important role in the production line of SiC. In this article, we illustrate each type of defect and their impact on device performance. We also put forward a thorough discussion about the pros and cons of different inspection technologies. The analysis presented in this review article not only provides an overview of various defect inspection techniques available for SiC but also helps researchers to make a wise choosing among these techniques in the context of industrial applications (Fig. 2). Table 2 lists the acronyms of the inspection techniques and defects in Fig. 2.

**Table 2:** The acronyms of the inspection techniques and defects in Fig. 2

SEM: Scanning electron microscopy	OM: Optical microscopy	BPD: Basal plane dislocation
DIC: Differential interference contrast	PL: Photoluminescence	TED: Threading edge dislocation
OCT: Optical coherence tomography	CL: Cathodoluminescence	TSD: Threading screw dislocation
XRT: X-ray topography	Raman: Raman spectroscopy	SF: Stacking faults

## Defects in SiC

**Fig. 3:**
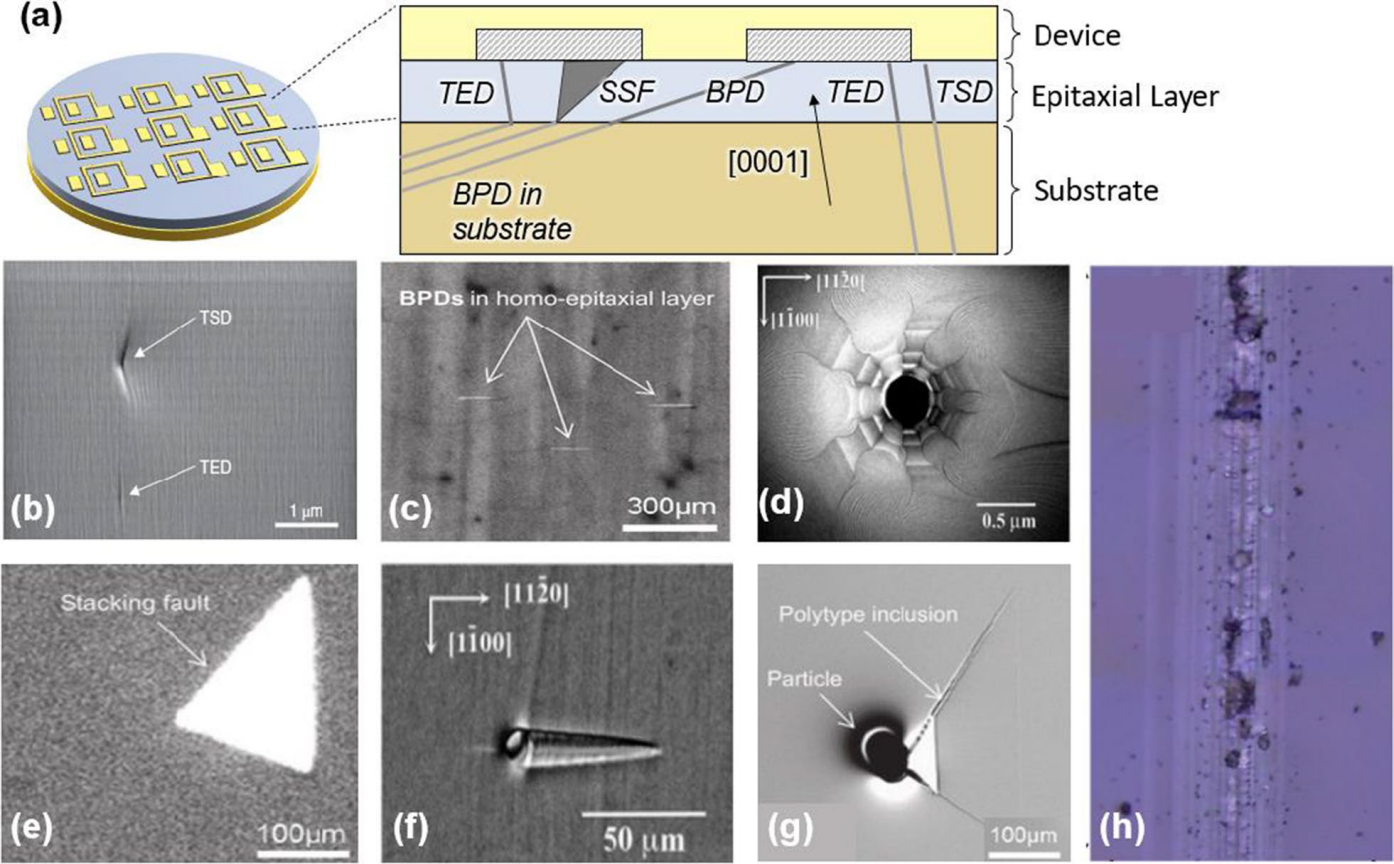
Various kind of defects appearing in SiC wafers. **a** Schematic cross-sectional view of SiC defects and image of **b** TEDs and TSDs [15], **c** BPDs [15], **d** Micropipes [16], **e** SFs [15], **f** carrot defects [16], **g** polytype inclusions [15], **h** Scratches [17]

Defects in SiC wafer are typically classified into two major categories: (1) crystallographic defects within the wafer and (2) surface defects at or near the wafer surface. As we further discuss in this section, crystallographic defects include Basal plane dislocations (BPDs), stacking faults (SFs), threading edge dislocations (TEDs), threading screw dislocations (TSDs), micropipes and grain boundaries, etc., as depicted in the cross-sectional schematic shown in Fig. 3a. Epitaxial layer growth parameters of SiC are very critical to the quality of wafer. Crystallographic defects and contaminations during growth processes [13] may extend into epitaxial layer and wafer surface to form various surface defects, including carrot defects, polytype inclusions, scratches, etc., or even convert to produce other defects [14], leading to detrimental effects on the final SiC devices.

The SiC epitaxial layers grown on 4° off-cut 4H-SiC substrate are the most common wafer type used today for a variety of device application. It is known that most of the defects are oriented parallel to the growth direction, therefore, epitaxial growth of SiC at an off-cut angle of 4° on SiC substrates not only preserves the underlying 4H-SiC crystal, but also allows the defects to have a predictable orientation. In addition, total number of wafers, that can be sliced from a single boule, increases. However, lower off-cut angle may generate other type of defects, such as 3C-inclusions and in-grown SFs [18–21]. In the coming subsections, we discuss the details about each type of defects.

### Crystallographic Defects

#### Threading Edge Dislocations (TEDs) and Threading Screw Dislocations (TSDs)

Dislocations in SiC are the main source for deterioration and failure of electronic devices [22–24]. Threading screw dislocations (TSDs) and threading edge dislocations (TEDs) both run along the [0001] growth axis with different Burgers vectors of <0001> and 1/3<11–20>, respectively. Both TSDs and TEDs may extend from the substrate to the wafer surface and bring about small pit-like surface features [15], as shown in Fig. 3b [25]. Typically, density of TEDs is about 8000–10,000 1/cm^2^, which is almost 10 times larger than that of TSDs. An extended TSD, where the TSD extends from the substrate to the epitaxial layer, may transform into other defects on the Basal plane and propagate along the growth axis during the SiC epitaxial growth. Harada et al. show that TSDs are converted to the stacking faults (SFs) or carrot defects on Basal planes during the SiC epitaxial growth [26], while TEDs in the epilayer are shown to be converted from BPDs inherited from the substrate during epitaxial growth.

#### Basal Plane Dislocations (BPDs)

Another type of dislocations is Basal plane dislocations (BPDs), which lie in the [0001]-plane of the SiC crystal with Burgers vector of 1/3<11–20>. BPDs rarely appear on the surface of SiC wafer [15]. These generally concentrate at the substrate with a density of 1500 1/cm^2^ while their density in the epitaxial layer is only about 10 1/cm^2^. Kamei et al*.* have reported that the density of BPDs decreases with increasing SiC substrate thickness [26]. BPDs show line-shaped features when using photoluminescence (PL) inspection, as shown in Fig. 3c [15]. An extended BPD may transform into SFs or TEDs during the SiC epitaxial growth.

#### Micropipes

The common dislocations observed in SiC are the so-called micropipes, which are hollow threading dislocations propagating along the [0001] growth axis with a large <0001> component of the Burgers vector. The diameter of micropipes ranges from a fraction of a micron to tens of microns. Micropipes show large pit-like surface features on the surface of SiC wafer [15]. Spirals which emanate from the micropipes, appearing as screw dislocations, are shown in Fig. 3d [16, 27]. Typically, the density of micropipes is around 0.1–1 1/cm^2^ and it continues to decrease in commercial wafers.

#### Stacking Faults (SFs)

Stacking faults (SFs) are defects with disarray of stacking sequence in SiC Basal planes. SFs may appear inside epitaxial layer by inheriting SFs in the substrate [15, 28, 29], or be associated with the transformation of extended BPDs and extended TSDs. Typically, the density of SFs is lower than 1 per cm^2^ and these show triangle-shaped features by using PL inspection, as shown in Fig. 3e [15]. However, various types of SFs can be formed in SiC, such as Shockley-type SFs and Frank-type SFs, etc., since just a small amount of stacking energy disorder between crystal plane may lead to considerable irregularities in the stacking sequence [30].

#### Point Defects

The point defect is formed by vacancy or interstitial of a single lattice site or a few lattice sites, which have no spatial expansion. Point defects can occur in every production process, especially in ion implantation. However, they are difficult to be detected and the interrelationship between point defects and the transformation of other defects is too complicated, which beyond the scope of this review.

#### Other Crystallographic Defects

There exist a few more type of defects in addition to those described in the above subsections. The grain boundary is a distinct boundary caused by the lattice mismatch between two different SiC crystal types when they intersect [31]. The hexagonal void is a crystal defect in which there is a hexagonal cavity within a SiC wafer and it has been proven to be one of the sources of micropipe defects that make high voltage SiC devices fail [32]. Particle inclusions are caused by downfall particles during growth process, and their density can be greatly reduced by proper cleaning, careful operation of pumping and control of gas flow procedures.

### Surface Defects

#### Carrots

Generally, surface defects are formed from extended crystallographic defects and contaminations. Carrot defect is a stacking fault complex with its length indicates the location of the TSD and SFs on Basal planes at both ends. The Basal faults are terminated by Frank partial dislocations, and the size of the carrot defect is related to the prismatic stacking faults [33]. The combination of these features forms the surface topography of a carrot defect, which resembles the shape of a carrot in its appearance with a density less than 1 per cm^2^, as shown in Fig. 3f [16]. Carrot defects are easily formed at polishing scratches, TSDs or an imperfection in the substrate [7].

#### Polytype Inclusions

Polytype inclusion, often referred to as triangular defect, is a lamellar inclusion of 3C-SiC polytype that extends to the surface of the SiC epilayer in a direction along Basal plane, as shown in Fig. 3g [15]. It may be generated by the downfall particles on the surface of the SiC epilayer during the epitaxial growth. As a result, the particle embedded in the epilayer and interferes with the growth process, creating a 3C-SiC polytype inclusion that shows acute-angled triangular surface feature with the particle located at the apex of the triangular region [15]. Many studies have also attributed the origin of polytype inclusions to surface scratches, micropipes and improper parameters of the growth process [34–36].

#### Scratches

Scratches are mechanical damages on the surface of SiC wafer formed during production processes, as shown in Fig. 3h [17]. Scratches on a bare SiC substrate may interfere with the growth of the epitaxial layer to create a high-density row of dislocations within the epitaxial layer, which are referred to as scratch traces [15], or scratches may become the basis for the formation of carrot defects [37]. Therefore, proper polishing of SiC wafers is critical, as scratches can have a significant impact on the device performance when these are present in the active region of the device.

#### Other Surface Defects

Step bunchings are surface defects formed during the SiC epitaxial growth and bring about obtuse-angled triangular or trapezoidal features on the surface of the SiC epilayer. There are many other surface defects such as surface pits, bumps and stain. These defects are usually created by non-optimized growth processes and incomplete removal of polishing damage, resulting in significantly detrimental impact on the performance of devices.

## Inspection Techniques

Quantifying the SiC substrate quality is an essential step before epitaxial layer deposition and device fabrication. After the epitaxial layer is formed, wafer inspection should be performed again to ensure that the location of defects is known, and their number is under control. Inspection techniques could be classified into surface inspection and subsurface inspection, depending on their ability to effectively extract structural information over or beneath the surface of the sample. As we further discuss in this section, in order to accurately identify the type of surface defects, KOH (potassium hydroxide) is usually used to visualize surface defects by etching them to a visible size under the optical microscope [38]. However, this is a destructive approach that cannot be used in in-line mass production. For in-line inspection, high-resolution non-destructive surface inspection techniques are required. Common surface inspection techniques include scanning electron microscopy (SEM), atomic force microscopy (AFM), optical microscopy (OM) and confocal differential interference contrast microscopy (CDIC), etc. For subsurface inspection, commonly used techniques include photoluminescence (PL), X-ray topography (XRT), mirror projection electron microscopy (MPJ), optical coherence tomography (OCT) and Raman spectroscopy, etc. In this review, we divide SiC inspection techniques into optical and non-optical methods and provide a discussion on each of techniques in the following sections.

### Non-optical Defect Inspection Technologies

Non-optical inspection techniques, those not involving any kind of optical probing, such as KOH etching and TEM, have been widely used for characterizing the quality of SiC wafers. These methods are relatively mature and precise to inspect defects on SiC wafers. However, these methods cause irreversible damage to the samples which then are not suitable for the use in the production lines. Although there exist other inspection methods like SEM, CL, AFM and MPJ which are non-destructive, the throughput of these methods is low and can serve as an assessment tool only. Next, we briefly introduce the principles of the above-mentioned non-optical technologies. Advantages and disadvantages of each individual technique are also brought under discussion.

#### Transmission Electron Microscopy (TEM)

The transmission electron microscopy (TEM) can be used to observe the subsurface structure of the sample at a nano-scale resolution. TEM makes use of accelerated electron beams incident onto the samples of SiC. Electrons with ultra-short wavelength and high energy pass through the surface of the sample which elastically scattered from the subsurface structure. Crystallographic defects in SiC, such as BPDs, TSDs and SFs, can be observed by using TEM [39–42].

**Fig. 4:**
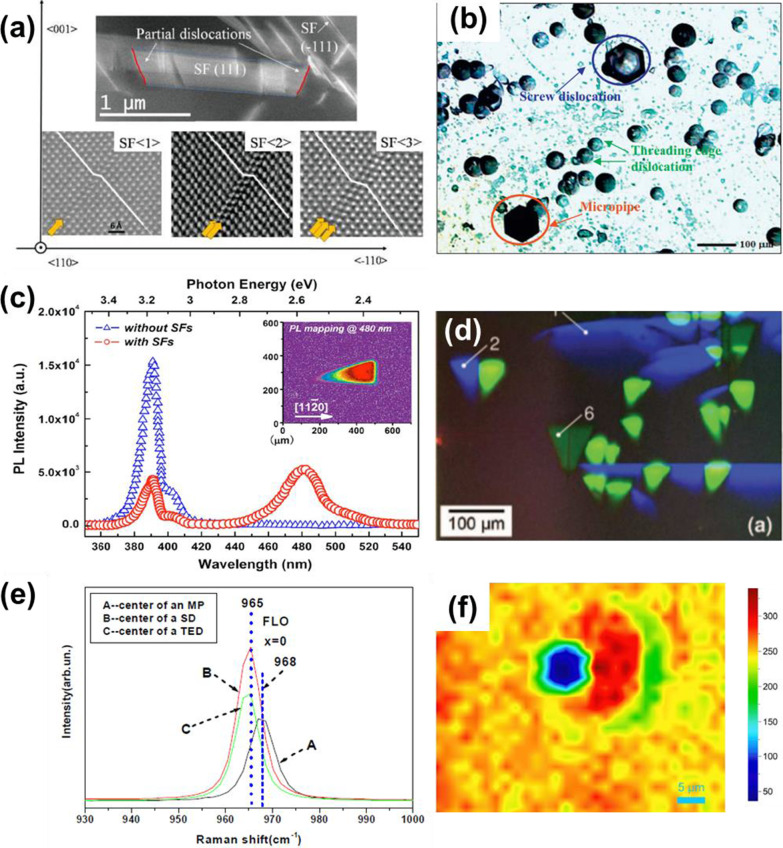
Different defect inspection methods and obtained images of defects. **a** TEM and HAADF image of SF [43]. **b**Optical micrograph image after KOH etching [45]. **c** PL spectrum with and without SF while the inset shows the monochromatic micro-PL mapping at a wavelength of 480 nm. [46]. **d** A real-color CL SEM image of SF at room temperature [47]. **e** Raman spectrum of various defects [48]. **f** Micro-Raman intensity map of the 204 cm^−1^ peak of a micropipe-related defect [49]

A scanning transmission electron microscope (STEM) is a type of transmission electron microscope, which can obtain atomic-level resolution through high-angle annular dark-field imaging (HAADF). Images obtained through TEM and HAADF-STEM are shown in Fig. 4a. A trapezoidal SF and partial dislocations are clearly visualized by the TEM image while the HAADF-STEM images show three kind of SFs observed in 3C-SiC. These SFs consist of 1, 2, or 3 faulted atomic layers, indicated by the yellow arrows [43]. Though TEM is a useful defect inspection tool, it can only provide one cross-sectional view at a time, so it takes a lot of time if one needs to inspect whole SiC wafer. Besides, mechanism of the TEM demands that the sample must be very thin, with a thickness of less than 1 μm, which makes preparation of the sample quite complicated and time-consuming [44]. Overall, TEM is used to understand the fundamental crystallography of defects, but it is not a practical tool for large scale or in-line inspection.

#### KOH Etching

KOH etching is another non-optical technique used to inspect defects of several kinds, such as micropipes, TSDs, TEDs, BPDs and grain boundaries. The patterns formed after KOH etching depend on experimental conditions such as etching duration and temperature of the etchant. When molten KOH at about 500℃ is added to SiC sample, it exhibits selective etching of SiC sample between areas with defects and those without defects in about 5 min [45]. After cooling and removing KOH from SiC sample, there are a lot of etched pits with different topography which are related to different types of defects. As shown in Fig. 4b, the dislocations produce large hexagonal etched pits assigned to micropipes, medium-sized pits to TSDs, and small-sized pits to TEDs. [45]

The advantage of KOH etching is that it can inspect all defects under the surface of SiC sample at one time, preparation of SiC sample is easy, and the cost is low. However, KOH etching is an irreversible process that can cause permanent damage to the sample. Further polishing of the sample is required to obtain a smooth surface after KOH etching.

#### Mirror Projection Electron Microscopy (MPJ)

Mirror projection electron microscopy (MPJ) is another promising subsurface inspection technique that allows the development of high throughput inspection systems capable of inspecting nanoscale defects. Since the MPJ reflects the equipotential image of surfaces on SiC wafers, the potential distortion caused by charged defects is distributed over a wider area than the actual defect size. Therefore, nanoscale defects can be inspected even the spatial resolution of the tool is in microscale. The electron beam from the electron gun passes through the focusing system and irradiates uniformly and normally onto the SiC wafer. Notably, the SiC wafer is irradiated by UV light, so the excited electrons are trapped by the defects present in the SiC wafer. Besides, SiC wafer is negatively charged to nearly equal to the acceleration voltage of the electron beam so that the incident electron beam decelerates and reflects before reaching the wafer surface. This phenomenon is similar to the reflection of light by a mirror, so the reflected electron beams are referred to as "mirror electrons." When the incident electron beam irradiates the SiC wafer carrying defects, the negatively charged state of the defect varies the equipotential surface, resulting in nonuniformity of the reflected electron beam. MPJ is a non-destructive inspection technique capable of imaging the static electrical potential topography on SiC wafers with high sensitivity. Isshiki et al. use MPJ to clearly identify BPDs, TSDs and TEDs after KOH etching [50]. Hasegawa et al. show images of BPDs, scratches, SFs, TSDs, and TEDs inspected by using MPJ [51] and discuss the relationship between latent scratches and step bunching [52].

#### Atomic Force Microscopy (AFM)

Atomic force microscopy (AFM) is generally applied to measure the surface roughness of SiC wafers with demonstrated resolution on atomic scale. The major difference between AFM and other surface inspection methods is that it does not suffer from diffraction limit of optical beams or aberration of lenses. AFM uses the interaction force between the probe tip on the cantilever and the surface of SiC wafer to measure the deflection of the cantilever which is then transduced into an electrical signal proportional to the characteristic appearance of the surface defects. AFM can form three-dimensional images of surface defects, but it is limited to resolve the topology of the surface and is time-consuming, so the throughput is low [53].

#### Scanning Electron Microscopy (SEM)

Scanning electron microscopy (SEM) is another non-optical technique used extensively for defect analysis of SiC wafers. SEM has high spatial resolution on the order of nanometers. A focused electron beam generated by accelerator scans the surface of SiC wafer and interacts with SiC atoms to produce various types of signals such as secondary electrons, back-scattered electrons and X-rays. SEM images corresponding to the output signal show the characteristic appearance of surface defects, which is useful for understanding structural information of SiC crystals. However, SEM is limited to surface inspection only and does not provide any information on sub-surface defects.

#### Cathodoluminescence (CL)

Cathodoluminescence (CL) spectroscopy makes use of focused electron beams to probe electronic transitions in a solid which results in the emission of characteristic light. CL facility usually comes with SEM because an electron beam source is common feature of the two technologies. Accelerated electron beams strike the SiC wafer and produce excited electrons. The radiative recombination of excited electrons emits photons with wavelengths in the visible spectrum. By combining the structural information and the functional analysis, CL gives a full description of a sample with direct correlation of a sample's shape, size, crystallinity, or composition with its optical properties. Maximenko et al. show the all-color CL image of SFs at room temperature, as displayed in Fig. 4d [47]. Different kinds of SFs corresponding to different wavelengths are apparent, and a common single-layer Shockley-type stacking fault with a blue emission at ~ 422 nm and a TSD at ~ 540 nm is found by the CL [47]. Although SEM and CL have a high-resolution owing to the electron beam source, the high energy electron beam might cause damage to the surface of the sample.

### Optical-Based Defect Inspection Technology

In pursuit of in-line mass production with high throughput without loss of inspection accuracy, optical-based inspection methods are promising because they can preserve the samples and most of them can provide rapid scanning capabilities. Surface inspection methods can be listed as OM, OCT and DIC, while Raman, XRT and PL are subsurface inspection methods. In this section, we describe the principles of each inspection method, how these apply in inspecting defects, and pros and cons of each method.

#### Optical Microscopy (OM)

The optical microscopy (OM), originally developed to closely view samples using light and optical magnifying components, can be utilized to inspect surface defects. This technique enables producing images in dark-field mode, bright-field mode, and phase mode, each giving specific defect information, and the combination of these images provides the ability to identify most of the surface defects [54]. When the inspection light illuminates on the surface of the SiC wafer, the dark-field mode captures the scattered light by surface defects, so the image has a dark background that excludes the unscattered light as well as bright objects that indicates the location of defects. On the other hand, the bright-field mode captures the unscattered light, showing a white background image with dark objects due to scattering of defects. The phase mode captures the images with phase shift, which are accumulated by the contamination on the surface of the SiC wafer, showing a phase-contrast image. The scattering image of OM is advantageous in lateral resolution, while the phase-contrast image mainly aims at examining the smoothness of the wafer surface. Several studies have made efficient use of optical microscopy to characterize surface defects. Pei Ma et al. show that very thin carrot defects or micropipe defects are too small to be inspected by optical coherence tomography (OCT) but can be examined by optical microscopy due to its advantages in lateral resolution [33]. Zhao et al. use OM to study the origin of polytype inclusions, surface pits and step-bunching [34].

#### Optical Coherence Tomography (OCT)

Optical coherence tomography (OCT) is an optical inspection techniques that can provide rapid, nondestructive and 3D subsurface images of investigated samples. Since OCT was originally applied for the diagnosis of many diseases, most of its applications have been to resolve images of biological and clinical biomedical samples. However, there has been a growing interest in applying OCT for inspection of defects in SiC wafers since the resolution of OCT has been improved to a sub-micron scale due to the development of advanced optical components for visible and infrared wavelengths. The light source used in OCT has a broadband spectrum consisting of a wide range of frequencies in the visible and infrared region, so the coherence length is small, which means that the axial resolution can be very high, while the lateral resolution depends on functionality of the optics. The principle of OCT is based on low-coherence interferometry, which is typically a Michelson type setup. The source light of the OCT is divided into two arms, a reference arm and an inspection arm. The light beam to the reference arm is reflected by a mirror, while the light beam to the inspection arm is reflected by the SiC wafer. By moving the mirror in the reference arm, the combination of the two light beams gives rise to interference, but only if the optical path difference between the two beams is less than a coherence length. Therefore, the interference signal acquired by the detector contains cross-sectional information of the SiC wafer, and by combining these cross-sectional inspections laterally, a 3D image of OCT can be achieved. However, the inspection speed and lateral resolution of OCT are still not comparable to other 2D inspection techniques, and the interference of surface scattering and absorption loss in the operating spectral range are the main limitations of OCT image formation. Pei Ma et al. use OCT to analyze carrot defects, polytype inclusions, grain boundaries and hexagonal voids [33]. Duncan et al. apply OCT to study the internal structure of single crystal SiC [55].

#### Differential Interference Contrast (DIC)

Differential interference contrast (DIC) is a microscopy technique that introduces phase contrast to the images of surface defects. The advantages of using DIC over OM are that the resolution of DIC is much higher than the phase mode of OM, because the image formation in DIC is not restricted by the aperture, and DIC can produce three-dimensional defect images by employing a confocal scanning system. The source light of DIC is linearly polarized by a polarizer and then split into two orthogonally polarized sub-beams, i.e., the reference beam and the inspection beam, by making it pass through a Wollaston prism. The reference beam strikes the normal surface of the SiC wafer, while the inspection beam strikes the surface of the SiC wafer with defects, producing a phase delay corresponding to the geometry of defects and alteration of optical path length. Since the two sub-beams are orthogonally polarized, they cannot interfere with each other during inspection until they are brought together after passing through a Wollaston prism again and enter an analyzer to generate defect-specific interference patterns. The processor then receives the defect signals to form a two-dimensional differential interference contrast image. To generate a three-dimensional image, a confocal scanning system can be used to shut off the two sub-beams that are offset from the focus of the system to avoid false inspections. Therefore, by making the focal point of the confocal system move in the direction of the optical axis, a three-dimensional defect image of the SiC wafer surface can be obtained. Sako et al. show that a surface defect with a scraper-shaped surface profile on the SiC epitaxial layer has been observed using CDIC. [56]. Kitabatake et al. establish the integrated evaluation platform using CDIC to inspect surface defects on the SiC wafers and the epitaxial films [57, 58].

#### X-Ray Diffraction Topography (XRT)

X-ray diffraction topography (XRT) is a powerful subsurface inspection technique that can help investigate the crystallographic structure of SiC wafers since the wavelength of X-rays is comparable with the distance between interatomic planes of SiC crystal. It is used to evaluate the structural characteristics of SiC wafers by measuring the change in diffraction intensity due to the strain field caused by defects. This means that crystallographic defects cause a change in lattice spacing or lattice rotation around them, forming a strain field. XRT is commonly used in production line with high throughput and sufficient resolution; however, it requires a large-scale apparatus for emission of X-rays and its defect mapping capabilities still require improvement. The image formation mechanism of XRT is based on Laue condition (momentum conservation), where a collimated beam of X-rays is produced when the electron beam generated by a heated filament is collimated and accelerated by a high electric potential to obtain sufficient energy, which is then directed to the metal anode. When X-rays are irradiated onto a SiC wafer, a unique diffraction pattern with several narrow and sharp peaks is formed and inspected by the detector due to the constructive and destructive interference of X-rays scattered at specific angles from the interatomic planes of SiC. Thus, crystallographic defects can be characterized by diffraction peak broadening analysis, where the diffraction spectrum is narrow and sharp if no defects are present; otherwise, the spectrum is broadened or shifted if there is a defect-induced strain field. The detection mechanism of XRT is based on X-rays diffraction rather than electrons scattering, thus classifying XRT as an optical technique while SEM is a non-optical technique. Chikvaidze et al. use XRT to confirm defects with different stacking sequence in the SiC sample [59]. Senzaki et al. show the transformation of extended BPDs to TED is origins of triangular-shaped single Shockley-type stacking fault (1SSF) inspected using XRT under current stress test [60]. Current in-line XRT is typically used to identify the defect structure without recognizable inspection signal from other inspection techniques such as PL and OM.

#### Photoluminescence (PL)

Photoluminescence (PL) is one of the most common subsurface inspection technique being used to inspect crystallographic defects. The high throughput of PL makes it suitable for in-line mass production. SiC is an indirect bandgap semiconductor that shows PL at near band-edge emission of about 380 nm wavelength. Recombination at through defect level in SiC wafers could be radiative. UV excitation-based PL technique has been developed to identify defects present inside SiC wafers, such as BPDs and SFs [61]. However, defects without characteristic PL features or with weak PL contrast against defect-free SiC region, such as scratches and threading dislocations, should be evaluated by other inspection methods. Since emission energy varies depending on the trap levels of defects, PL images with spectral resolution could be used to differentiate each type of defects and map them [15]. The PL spectrum of polytype SF exhibits multi-peak spectra in the wavelength range of 350–550 nm due to the quantum-well-like band structure induced by SFs. Each type of SF can be distinguished by examining their emission spectra using bandpass filter that filters out individual spectra, as shown in Fig. 4c [15, 46]. Berwian et al. construct a defect luminescence scanner based on UV-PL to clearly inspect BPDs, SFs and polytype inclusions [62]. Tajima et al. use PL with a variety of excitation wavelengths ranging from deep UV to visible and NIR to inspect TEDs, TSDs, SFs and examine the correlation between the PL and etched pit patterns [63]. Nevertheless, the PL images of some defects are similar, such as BPDs and carrot defects, which both show line-shaped features, making it difficult for PL to distinguish between them, so other structural analysis tools, such as XRT or Raman spectroscopy, are often used in parallel with PL to accurately classify these defects.

#### Raman Spectroscopy

Raman spectroscopy has a wide variety of applications in biology, chemistry and nanotechnology to identify features of molecules, chemical bonds and nanostructures. Raman spectroscopy is a non-destructive subsurface inspection method that can verify different crystalline structures and crystallographic defects in SiC wafers [64, 65]. Typically, the SiC wafer is irradiated by a laser and the laser light interacts with molecular vibrations or phonons in the SiC that puts the molecule into a virtual energy state, resulting in an upward or downward shift in the wavelength of the inspected photons, referred to as Stokes Raman scattering or Anti-Stokes Raman scattering, respectively. The shift in the wavelength gives information about the vibrational modes in SiC, corresponding to the different polytype structures. It has been shown that the characteristic peaks at 200 and 780 cm^−1^ in the measured Raman spectrum indicate the 4H-polytype of SiC, while the characteristic peaks at 160, 700 and 780 cm^−1^ represent the 6H-polytype of SiC [66]. Chikvaidze et al. use Raman spectroscopy to confirm a 2H-SiC polytype with Raman peaks around 796 and 971 cm^−1^ present in the 3C-SiC sample [67]. Hundhausen et al. use Raman spectroscopy to study the polytype conversion of 3C-SiC during high-temperature annealing [68]. Feng et al. find the peak center shift and the intensity variation of micropipes, TSDs and TEDs, as shown in Fig. 4e [48]. For spatial information, an image of Raman mapping is shown in Fig. 4f [49]. Generally, the Raman scattering signal is very weak, so it takes a long time for Raman spectroscopy to collect sufficient signal. The technique could be used for detail analysis of the defect physics, but it is not suitable for in-line inspection due to weak signal and current technology limits.

The types of defects that can be inspected by the inspection methods discussed in this paper and their corresponding researches are summarized in Table 3, which still requires more research data to be completed.

**Table 3:** Research on various inspection methods and inspected defect types

	Non-optical						Optical				
	Inspection			Metrology			Inspection		Metrology		
	KOH	SEM	AFM	TEM	CL	MPJ	OCT	DIC	XRT	PL	Raman
*Crystal defect*											
Micropipe	[45]	[16]	[69]	[70]	[71]	X	O	O	[72]	[15]	[48]
TSD/TED	[45, 46, 73]	[73]	O	[74]	[47]	[51, 73]	O	O	[16, 75]	[15, 61, 76]	[48]
BPD	[46, 73]	X	X	[74]	[77]	[51, 73]	X	X	[75]	[15, 61]	O
SF	[78]	[16]	O	[43, 46, 74]	[47]	[51]	O	O	[76]	[15, 61, 46]	[17]
*Surface defect*											
Scratch		[79]	[79]		O	[51, 79]	O	O	[15, 80]	X	[17]
Carrot		[16]	O		[81]	[82]	[33]	[82]	[83]	[15]	O
Triangular		[16]	[25]		[71]	O	[33]	[84]	[84]	[85]	[84–86]
Downfall		[87]	O		[87]	X	O	O	X	X	O

## Impact of Defects on Devices

Each type of defect adversely affects the quality of the wafer and deteriorates the devices subsequently fabricated on it. The deterioration between defects and device failures is related to the kill ratio, which defined as the proportion of defects estimated to cause device failure. The kill ratio for each defect type varies depending on the end application. Specifically, those defects that cause significant impact on the device are referred to as killer defects [88]. Previous studies have shown the correlation between defects and device performance [89, 90]. We discuss the impact of different defects on different devices in this section.

In MOSFET, BPDs increase on-resistance [91] and reduce the gate oxide reliability [92]. Micropipes limit the operation current and increase the leakage current [93, 94] while defects such as SFs, carrots and polytype inclusions reduce blocking voltage [4, 91] and scratches on the surface cause reliability issues [95]. Isshiki et al. show that there are latent scratches, consisting of complex stacking faults and dislocation loops lying beneath the SiC substrate, resulting in formation of step bunching and degradation of dielectric strength of oxide film in SiC-MOSFETs [79]. Other surface defects such as trapezoidal features might lead to significant impact on the channel mobility or the oxide breakdown characteristics in SiC MOSFETs [96].

In Schottky barrier diode, BPDs, TSDs and TEDs increase the reverse leakage current [97–101] while micropipes and SFs reduce the blocking voltage [85, 89, 102]. Carrots and polytype inclusions both reduce blocking voltage and increase leakage current while scratches cause barrier height inhomogeneity [103].

In a *p*–*n* diode, BPDs increase the on-resistance and leakage current [91] while TSDs and TEDs reduce blocking voltage [104]. Micropipes limit the operation current and increase the leakage current [93, 94] while SFs increase forward voltage [105]. Carrots and polytype inclusions reduce blocking voltage and increase leakage current [72, 106] while scratches on the surface have no direct impact on *p*–*n* diode. Skowronski et al. show that during the diode operation, the BPD within the SiC epitaxial layer is transformed into a SFs or allows the SFs to extend along the BPD through electrical conduction, resulting in current degradation that increases the resistance of the SiC *p*–*n* diode [60]. Studies have also proved that the SFs may give rise to a 3C-SiC polytype, resulting in decrease in minority carrier lifetime of the SiC *p*–*n* diode because the 3C-SiC polytype has a lower bandgap than the 4H-SiC polytype, so a SF act as a quantum well that enhances the recombination rate [107]. Moreover, the single Shockley-type SFs are expanded under PL characterization, causing a change in junction potential which in turn deteriorates the on-resistance of SiC *p*–*n* diode [108]. Furthermore, TSDs result in degradation of the blocking voltage and TEDs reduce the minority carrier lifetime of the SiC *p*–*n* diode [109].

In bipolar devices, BPDs reduce the gate oxide reliability [51, 110] while TSDs and TEDs reduce carrier lifetime [111]. Micropipes limit the operation current [94] while SFs reduce carrier lifetime [111]. Carrots and polytype inclusions reduce blocking voltage and increase leakage current and reduce carrier lifetime [84, 112].

Point defects (vacancies) in SiC reduce the carrier lifetime of the device [24], leading to junction leakage currents [113] and resulting in lower breakdown voltages. Although point defects have a negative impact on electronic devices, they find some useful applications as well, such as in quantum computing [114, 115]. Lukin et al. show that point defects in SiC such as silicon vacancy and carbon vacancy can produce stable bound states with suitable spin–orbit attributes, serving as hardware platform choices for quantum computation [116].

**Fig. 5:**
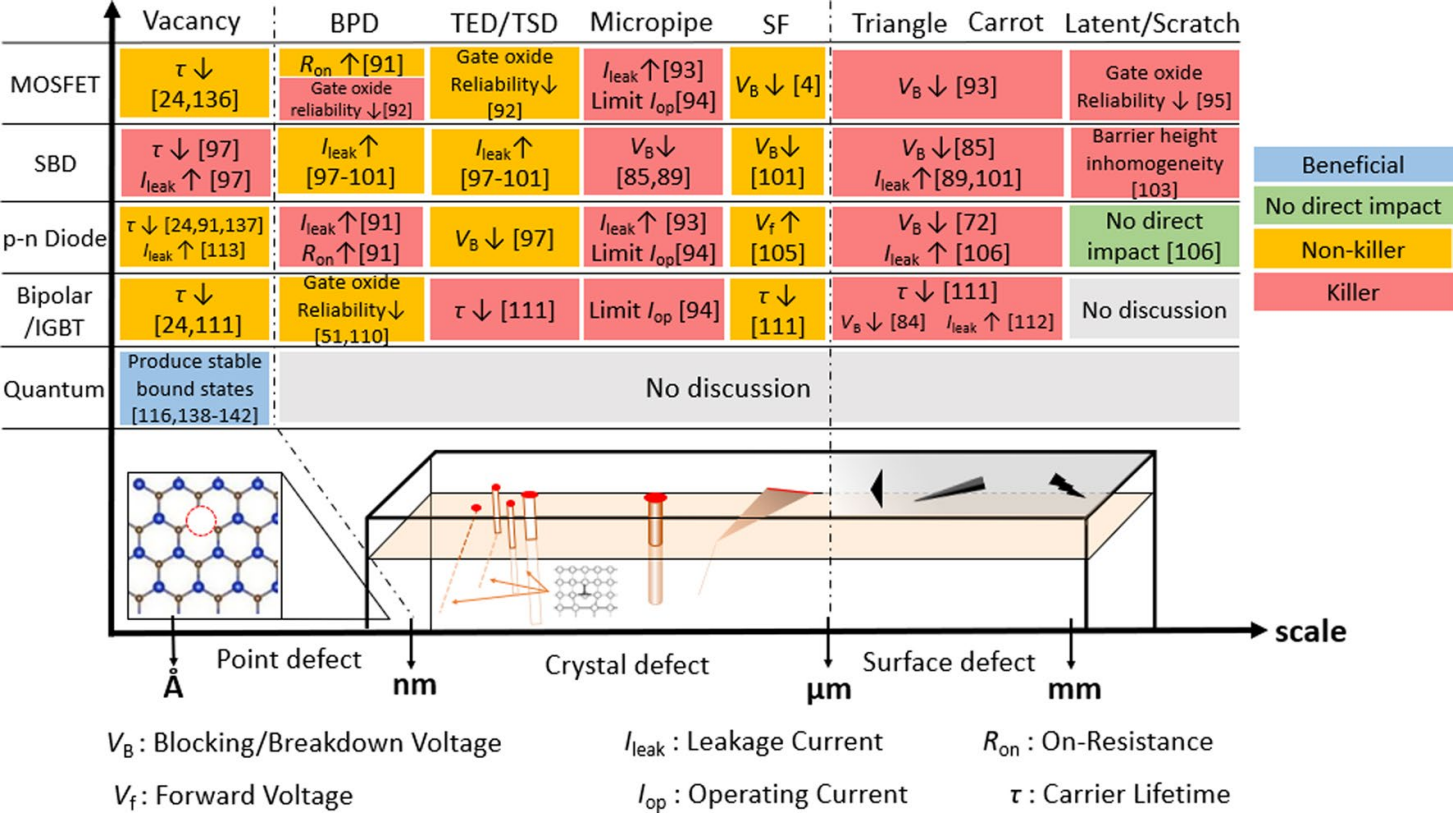
The impact of defects on different devices

**Fig. 6:**
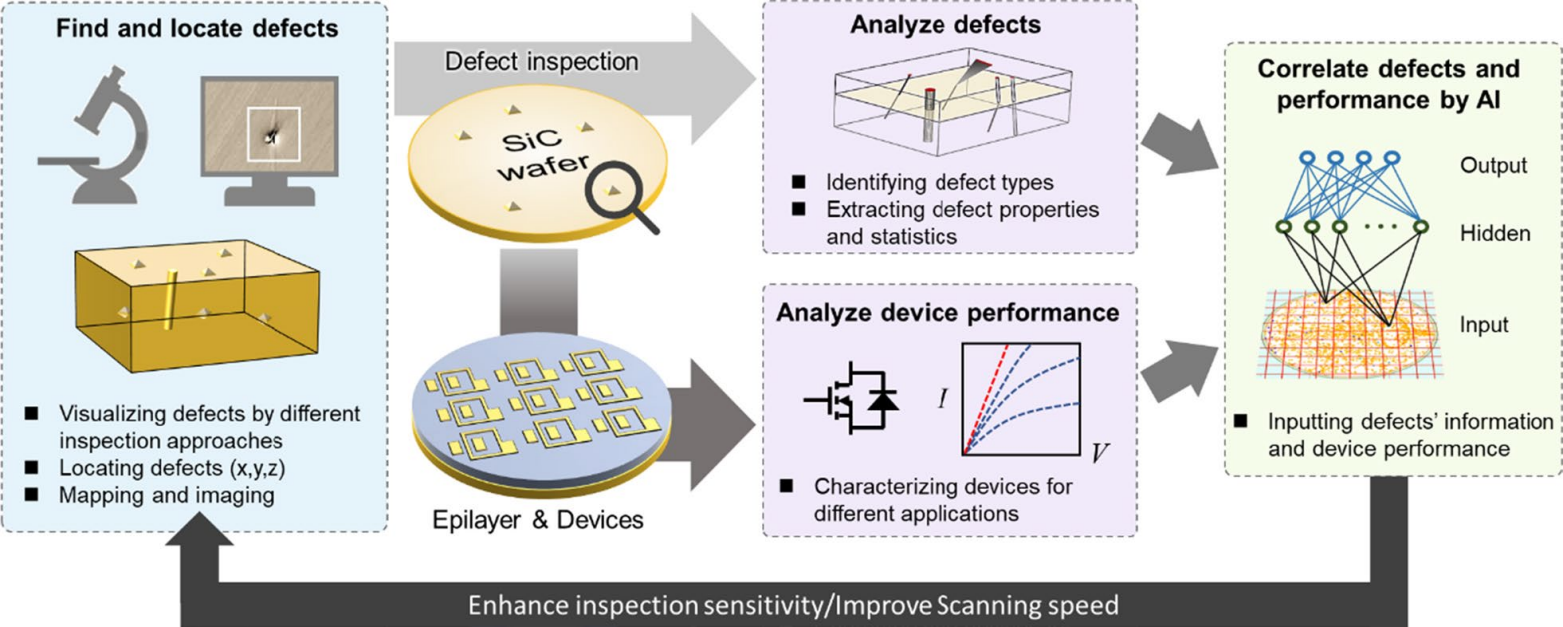
Defect detection and device performance estimation assisted by AI

**Fig. 7:**
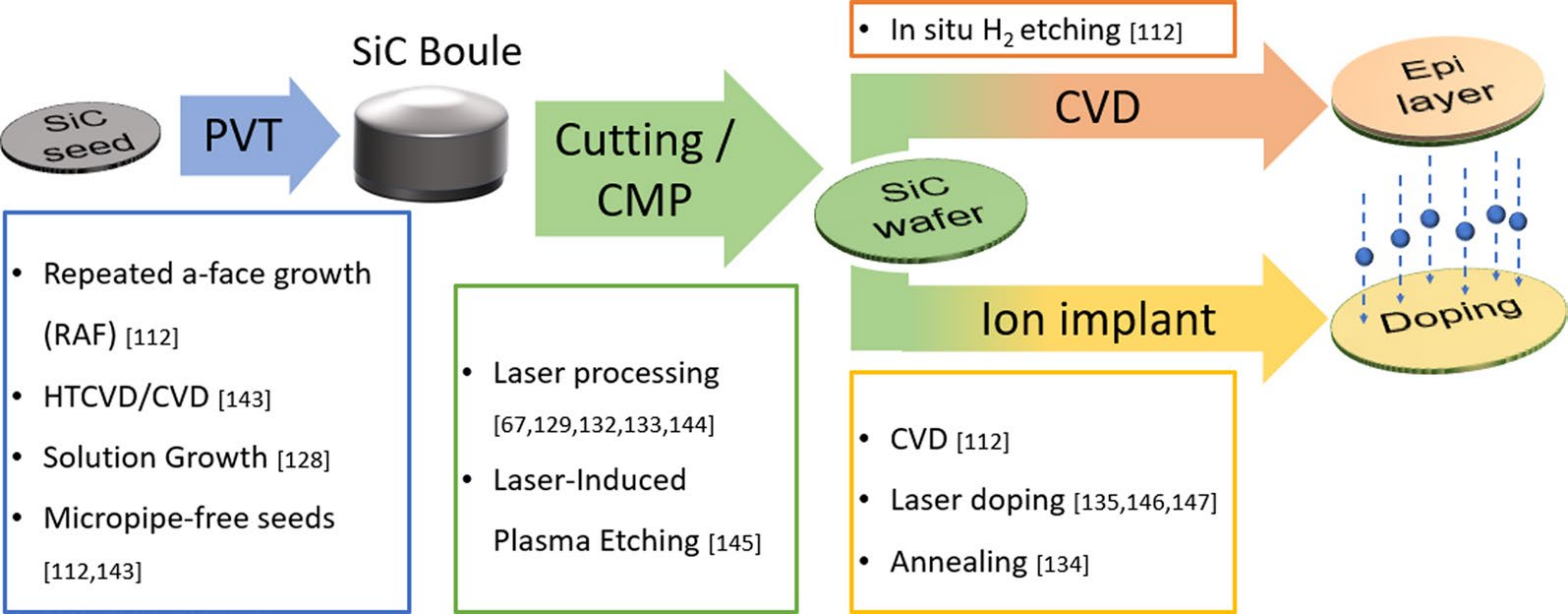
Method of using laser to reduce defects in the manufacturing process

The impacts of defects on different devices are organized in Fig. 5. As one can see, defects can deteriorate the device characteristics in many ways [91, 117]. Although the negative effects of defects can be counteracted by designing different device structures [1–3, 118–123], establishing a fast and accurate defect inspection system is amid a pressing need to help one observe defects and further optimize the process to reduce them. Note that analyzing the characteristics of SiC devices to identify type and the presence of defects could potentially be used as a defect inspection method (Figs. 6 and 7).

An efficient defect inspection system requires the ability to identify surface defects and crystallographic defects simultaneously, put all defects to the correct category and then display the mapping of defects distribution data of the entire wafer by using multi-channel machine learning algorithms. Kawata et al. design an automatic inspection algorithm for the dislocation contrasts of *n*-type SiC wafers in a birefringence image and succeed in inspecting the position of the dislocation contrasts of XRT images with relatively high precision and sensitivity [124]. Leonard et al. use deep convolutional neural network (DCNN) machine learning for automated defect inspection and classification by using PL images of unetched wafers coupled with automatically labelled images of the corresponding etched wafers as the training set. The defect locations and classifications determined by DCNN correlate well with the subsequently etch delineated features [125]. Monno et al. propose a deep learning system which inspecting defects on SiC substrate by SEM and classifying them with a 70% accuracy. The proposed approach can combine multiple tiles without inconsistency of linear defects and can inspect and classify the seven defects, with a very good degree of accuracy [126, 127].

Apart from inspecting defects, reducing their density is also a useful approach to improve the quality and the yield of SiC devices. By using micropipe-free seeds or a solution-based growth, the density of micropipe and TSDs can be decreased [112, 128]. To reduce the surface defects caused by mechanical processes, some studies point out that femtosecond lasers can be used to improve the efficiency of chemical–mechanical planarization [129] and the cutting quality [67, 130–133]. Femtosecond laser annealing can also improve the quality of ohmic contact between Ni and SiC and increase the conductivity of the device [134]. In addition to the application of femtosecond lasers, some other teams also found that the use of laser-induced liquid phase doping (LILPD) can effectively reduce the damage generated during the process [135].

## Conclusion

In this review article, we described the importance of defect inspection in the SiC industry, especially of those known as killer defects. Details of the crystallographic and surface defects that often arise during the production of SiC wafers as well as the nature of deterioration caused by these defects in different devices are comprehensively reviewed. Surface defects are detrimental to most devices, while crystallographic defects are risky for defect transformation and wafer quality. After understanding the impact of defects, we summarize the principles of common surface and subsurface inspection techniques, how these are applied in inspecting defects, and pros and cons of each method. The destructive inspection techniques can provide observable, reliable, and quantitative information; however, these cannot meet the requirements of in-line mass production since these are time consuming and adversely affect the quality of sample. On the other hand, non-destructive inspection techniques, especially optical-based techniques, are more applicable and efficient in production line. Note that different inspection techniques are complementary to each other. A combinational use of inspection techniques could potentially balance the tradeoff between throughput, resolution and equipment complexity. In the future, it is anticipated that non-destructive inspection methods with high-resolution and rapid scanning capabilities are integrated into the perfect defect inspection systems capable of simultaneously inspecting surface defects and crystallographic defects, then using multi-channel machine learning algorithms to assign all defects to the correct category and display the mapping image of defect distribution data to the entire SiC wafer.

## Data Availability

Not applicable.

## Abbreviations

SiC: Silicon carbide
Si: Silicon
GaAs: Gallium arsenide
GaN: Gallium nitride
MOSFET: Metal–oxide–semiconductor field-effect transistor
SBD: Schottky barrier diode
IGBT: Insulated gate bipolar transistor
PVT: Physical vapor transport
CMP: Chemical–mechanical planarization
CVD: Chemical vapor deposition
Epi layer: Epitaxial layer
KOH: Potassium hydroxide
TEM: Transmission electron microscopy
STEM: Scanning transmission electron microscope
HAADF: High-angle annular dark-field imaging
SEM: Scanning electron microscopy
AFM: Atomic force microscopy
XRT: X-ray topography
XRD: X-ray diffraction
Raman: Raman spectroscopy
PL: Photoluminescence
MPJ: Mirror projection electron microscopy
OCT: Optical coherence tomography
OM: Optical microscopy
CL: Cathodoluminescence
DIC: Differential interference contrast
C-DIC: Confocal differential interference contrast microscopy
BPD: Basal plan dislocation
SF: Stacking faults
TED: Threading edge dislocation
TSD: Threading screw dislocation
APDs: Anti-phase domains
BSDs: Black spot defects
DCNN: Deep convolutional neural network
LILPD: Laser-induced liquid phase doping
HTCVD: High-temperature chemical vapor deposition
